# X-ray fluorescence mapping of brain tissue reveals the profound extent of trace element dysregulation in stroke pathophysiology

**DOI:** 10.1093/mtomcs/mfae054

**Published:** 2024-11-15

**Authors:** M Jake Pushie, Nicole J Sylvain, Huishu Hou, Nicole Pendleton, Richard Wang, Liam Zimmermann, Maxwell Pally, Francisco S Cayabyab, Lissa Peeling, Michael E Kelly

**Affiliations:** Division of Neurosurgery, Department of Surgery, College of Medicine, University of Saskatchewan, Saskatoon, SK S7N 5E5, Canada; Division of Neurosurgery, Department of Surgery, College of Medicine, University of Saskatchewan, Saskatoon, SK S7N 5E5, Canada; Division of Neurosurgery, Department of Surgery, College of Medicine, University of Saskatchewan, Saskatoon, SK S7N 5E5, Canada; Division of Neurosurgery, Department of Surgery, College of Medicine, University of Saskatchewan, Saskatoon, SK S7N 5E5, Canada; College of Medicine, Department of Medicine, University of Saskatchewan, Saskatoon, SK S7N 5E5, Canada; College of Medicine, Department of Medicine, University of Saskatchewan, Saskatoon, SK S7N 5E5, Canada; College of Arts & Science, Anatomy, Physiology and Pharmacology, University of Saskatchewan, Saskatoon, SK S7N 5E5, Canada; Division of Neurosurgery, Department of Surgery, College of Medicine, University of Saskatchewan, Saskatoon, SK S7N 5E5, Canada; Division of Neurosurgery, Department of Surgery, College of Medicine, University of Saskatchewan, Saskatoon, SK S7N 5E5, Canada; Division of Neurosurgery, Department of Surgery, College of Medicine, University of Saskatchewan, Saskatoon, SK S7N 5E5, Canada

**Keywords:** X-ray fluorescence microscopy, brain metals, trace element imaging, ischaemia, stroke

## Abstract

The brain is a privileged organ with regard to its trace element composition and maintains a robust barrier system to sequester this specialized environment from the rest of the body and the vascular system. Stroke is caused by loss of adequate blood flow to a region of the brain. Without adequate blood flow ischaemic changes begin almost immediately, triggering an ischaemic cascade, characterized by ion dysregulation, loss of function, oxidative damage, cellular degradation, and breakdown of the barrier that helps maintain this environment. Ion dysregulation is a hallmark of stroke pathophysiology and we observe that most elements in the brain are dysregulated after stroke. X-ray fluorescence-based detection of physiological changes in the neurometallome after stroke reveals profound ion dysregulation within the lesion and surrounding tissue. Not only are most elements significantly dysregulated after stroke, but the level of dysregulation cannot be predicted from a cell-level description of dysregulation. X-ray fluorescence imaging reveals that the stroke lesion retains <25% of essential K^+^ after stroke, but this element is not concomitantly elevated elsewhere in the organ. Moreover, elements like Na^+^, Ca^2+^, and Cl^−^ are vastly elevated above levels available in normal brain tissue (>400%, >200%, and >150%, respectively). We hypothesize that weakening of the blood–brain barrier after stroke allows elements to freely diffuse down their concentration gradient so that the stroke lesion is in equilibrium with blood (and the compartments containing brain interstitial fluid and cerebrospinal fluid). The change observed for the neurometallome likely has consequences for the potential to rescue infarcted tissue, but also presents specific targets for treatment.

## Graphical Abstract

**Graphical Abstract:**
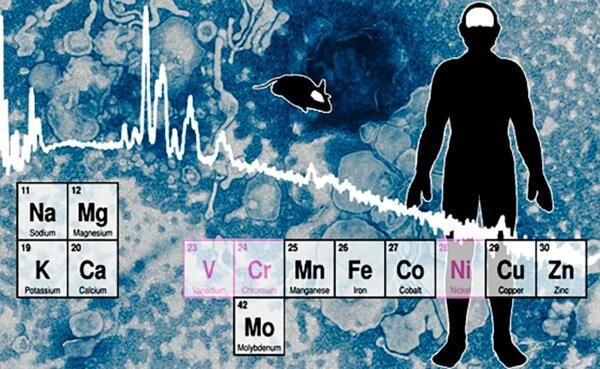

## Introduction

Emerging interest in elemental mapping techniques in the biomedical sciences has been matched with methodological advances [1–4] including synchrotron-based methods and off-line (non-synchrotron source) instruments—such as the AttoMap (Sigray, USA) or laser ablation inductively coupled plasma mass spectrometry (LA-ICP-MS) [4, 5]. Our research program focuses on brain metabolism and stroke in mouse models as a surrogate for human brain tissue. Studies of animal models offer greater control over environmental and biological variables, and while organism- and system-level differences are inescapable much of the underlying pathophysiology and cell-level processes are analogous, if not identical [6–8]. Herein, we demonstrate ion and trace element dyshomoeostasis in the brain after stroke onset. We highlight a shift in perspective regarding the trace element changes in relation to the dogma of stroke pathophysiology. We demonstrate that trace elements are more profoundly dysregulated following stroke and that these changes transcend the local changes occurring at the cell level.

Stroke contributes to millions of deaths and leaves further millions permanently disabled every year [9]. Stroke arises due to loss of adequate blood flow to a region of the brain, resulting in ischaemia. If ischaemic conditions persist then it can result in cell death within minutes. While living in a developed country is often a predictor for improved health and reduced risk for many diseases, cerebrovascular accidents remain a leading cause of death around the world, regardless of socioeconomic status [10]. The neurometallome is maintained to ensure the continued functioning of the brain. Metabolic dysregulation, such as occurs in stroke, triggers an ischaemic cascade that results in ion dysregulation, loss of function, increased cell toxicity, and oxidative damage [11].

The brain is a privileged organ that maintains a specific complement of ions and trace elements compared to most other organs, particularly in comparison with blood. The brain is protected from the external environment of the body through the blood–brain barrier and blood–cerebrospinal fluid (CSF) barrier. The CSF, for example, contains similar concentrations of ions as those in arterial plasma (Table 1), which are in contrast to the concentrations found within cells that are abundant in the brain parenchyma [12]. The neurons and astrocytes in the brain contain significantly elevated K^+^ compared with blood or CSF, although the intracellular concentration of this ion is similar to that found in muscle cells [13]. The brain also contains relatively elevated Fe, Cu, and Zn compared with blood and other organ systems. Additional transition metals important for mammalian systems, including homoeostasis and functioning of the brain, include Mn and Mo, although these elements are maintained at particularly low levels compared to more abundant metals in the brain [14]. Beyond the transition metals the brain also maintains tight control over Na^+^, Mg^2+^, K^+^, and Ca^2+^—unlike the transition metal ions the charge is specified for these elements due to the octet rule for outer valence orbital filling, which makes the cationic forms the most stable. With the exception of Zn^2+^, due to the filled *d*-orbital shell (*d*^10^), the transition metal ions often have more than one charge state that is accessible, allowing these elements to partake in electron transfer reactions [15]. Unlike the metals Na^+^, Mg^2+^, K^+^, and Ca^2+^, which are required in significant concentrations for biological functioning, the transition metals typically serve specialized roles within small molecules, in the active sites of enzymes, or as structural components in larger biomolecules. As a result, the transition metals are almost always maintained at lower levels in cells and tissues compared with bulk ions like Na^+^ or Cl^−^. These ions are not only absolutely essential for normal brain function, but are also tightly controlled in the brain and CSF, with K^+^ serving as an exemplary case, demonstrating insignificant changes in concentration despite large perturbations in total body levels [16]. Of the non-metals essential for life, the importance of C, H, O, N, P, and S is a foregone conclusion. The non-metals Cl^−^, Se, and I^−^ are also essential, although only Cl^−^ is maintained in significant quantities in the brain, while Se and I^−^ exist at low trace levels [14, 17].

**Table 1.** Selected elements of the neurometallome in essential compartments^a^

**Element**	**Neuron**	**Astrocyte**	**CSF**	**Blood plasma**
Na^+^	10	55	147	150
Mg^2+^	8	*n/a*	1.15	0.86
K^+^	125	80	3	4
Ca^2+^	0.75	*n/a*	1.14	2.5
Cl^−^	6.6	30	120	100
Zn^2+^	0.35	*n/a*	15 ×* *10^−8^	14 ×* *10^−6^

^a^Total concentrations are listed in mmol/L [12]. Some astrocyte data are not available.

**Figure 1.**
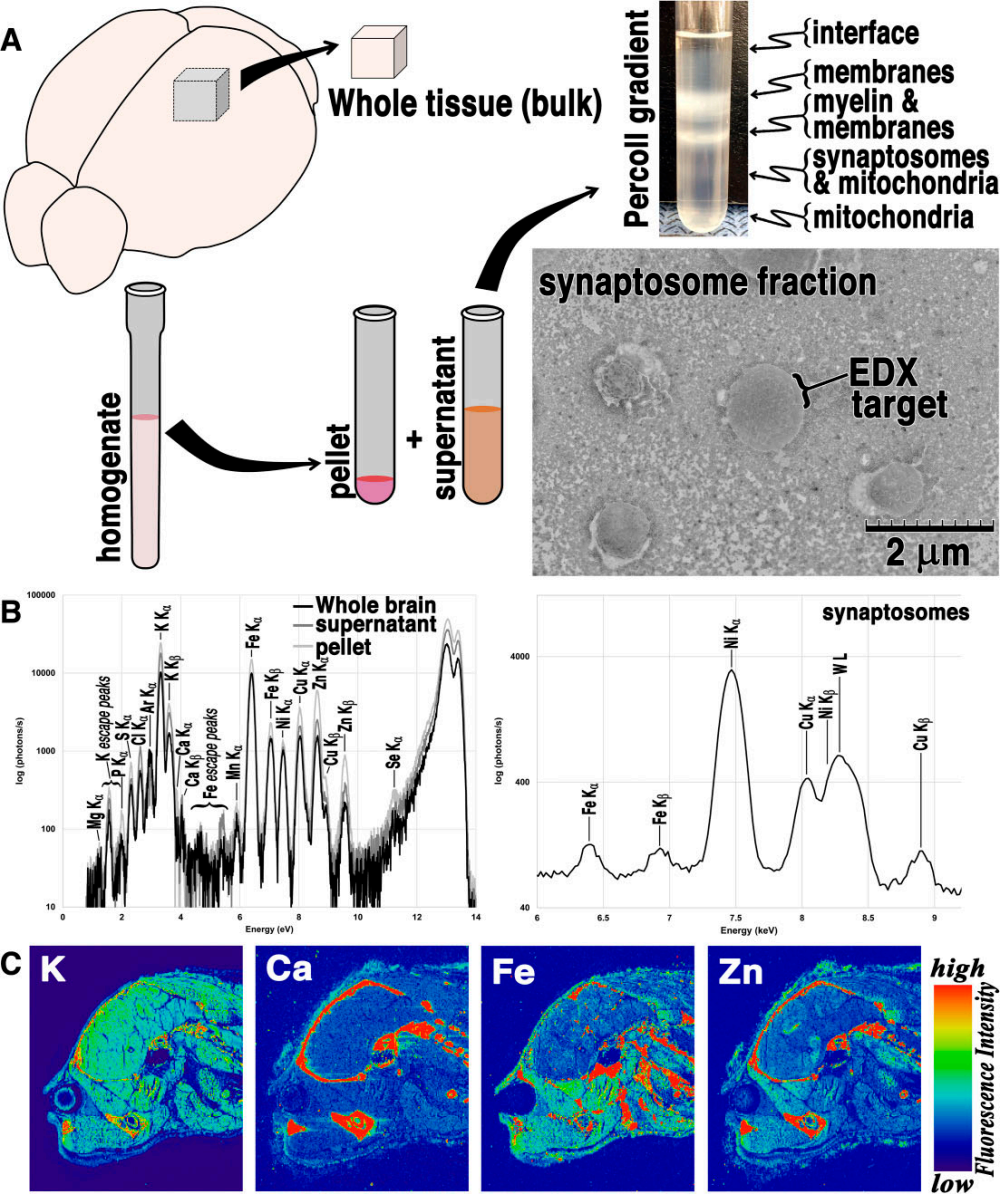
(A) Schematic depiction of brain tissue preparation for bulk analysis and isolation of subcellular components (i.e. membrane fragments, synaptosomes, and mitochondria. (B) X-ray fluorescence emission spectrum from whole brain tissue, compared with isolates from centrifugation of brain homogenate. The second X-ray fluorescence spectrum is obtained from targeted EDX detection of a purified synaptosome (the EDX target shown in the TEM image in panel B). The Ni fluorescence in the probed synaptosome is due to the Ni-grid used to mount the specimen. (C) A sagittal view through the head of a mouse, demonstrating the elemental distribution of the brain *in situ* (full body shown in Supplementary Fig. S2).

The techniques for characterizing element levels and distributions in biological systems can generally be divided into bulk analysis and elemental distribution methods. Bulk analysis includes any technique that characterizes the elemental content in a specimen without taking into account where the elements are localized in the sample. Examples of this include X-ray absorption and X-ray emission-based spectroscopies [18, 19], atomic absorption methods [20, 21], Particle-induced X-ray emission (PIXE) [22], and ICP-MS [23]. Figure 1A demonstrates the preparative steps in bulk analysis of brain tissue in contrast to imaging data from intact tissues (Fig. 1C). Mapping the distribution of elements within specimens has advanced considerably in the past two decades, and their applications to biological systems have been highlighted in a number of overview articles for techniques such as X-ray fluorescence imaging (XFI) [4, 5] (e.g. Fig. 1C), K-edge subtraction (KES) [24, 25], and LA-ICP-MS [26, 27].

Herein, we employ X-ray-based techniques to excite multiple elements simultaneously through X-ray absorption. X-ray absorption promotes an inner shell electron from its ground state orbital to a higher energy orbital. Once in the excited state, relaxation back to the ground state results in emission of an X-ray photon with an energy characteristic of the orbitals (and element) involved in the emission [4, 5]. These techniques have been employed to characterize ion dysregulation in representative examples of mouse stroke models, which serve as exemplars of trace element dysregulation after stroke injury. The distribution of elements in the healthy brain is presented as well as an overview of membrane trafficking at the blood–brain barrier, in the context of stroke. Representative stroke specimens demonstrate the significant metabolic dysregulation observed post-stroke, as probed with XFI and Fourier transform infrared (FTIR) imaging. Additional imaging data are contained in the Supplementary data accompanying this article.

## Materials and Methods

### Animal stroke models

Eleven-week-old male and female C57BL/6 mice were housed with a 12-h light/dark cycle with *ad libitum* access to chow and water. Animals were anaesthetized using isoflurane (5% induction, 1.5%–2% maintenance in 40/60 O_2_/N_2_O, Baxter Corporation, Toronto, ON, Canada), and the head was secured in a stereotaxic frame. Body temperature was monitored and maintained throughout the procedures using a RightTemp® temperature monitor with homoeothermic controller (Kent Scientific Corporation, USA).

Photothrombotic stroke mice had the fur at the top of the skull removed using a handheld shaver (Braun Cruzer, Procter & Gamble, Canada) and were administered an intraperitoneal injection of Rose Bengal (100 mg/kg; Sigma, USA), 5 min prior to subsequent steps. A sterile metal mask was used on the area surrounding the primary somatosensory cortex to minimize laser light scatter, and the S1FL region of the cortex was illuminated for 20 min with a 532-nm laser (24.2 mW) that was located 3 cm above the skull. This duration was sufficient to activate the Rose Bengal in circulation and induce a focal thrombus. The time of laser shut-off is assumed to be the post-stroke time zero. Animals were maintained until 1 or 72 h post-stroke. Control (sham) surgeries employed all of the same steps, except the laser was not powered-on. After procedures, bupivacaine (2 mg/kg, administered at 1.67 mg/ml in saline) was applied to the open incision site prior to suturing.

The middle cerebral artery occlusion (MCAO) mouse model followed established methods and was initiated by making a midline incision in the neck and the tissue reflected laterally to reveal the common carotid artery (CCA), which is relatively superficial in C57BL/6 mice. The CCA is traced to locate the internal and external carotid arteries (ICA and ECA, respectively), and the proximal end of the CCA is ligated with 6-0 braided suture material, as well as the ECA branch. Loose-fitting sutures are placed distally around the CCA and at the ICA. An incision is made in the CCA, between the two CCA sutures, and a flexible plastic filament is inserted through the incision and advanced into the ICA, with the CCA and ICA sutures kept loose enough to allow the filament to pass through. The filament is advanced through the circle of Willis until the middle cerebral artery (MCA) is occluded and then the loose-fitting CCA and ICA sutures are tightened to maintain placement of the filament. The filament occludes the MCA for 30 min and is then withdrawn, followed by tightening of the distal CCA. Sutures on the ICA and ECA are finally removed. The post-occlusion reperfusion time is assumed to start once the filament is removed. Bupivacaine (1 mg/kg at 1 mg/ml) is dripped into the incision and the skin is sutured using 4-0 nylon monofilament. Control (sham) surgeries followed all of the same steps except the filament was removed immediately after advancement toward the MCA. MCAO animals receive post-operative buprenorphine and a subcutaneous injection of saline (10 ml/kg per hour of surgery). All animals in the current study were maintained until 60 or 90 min post-reperfusion.

Intracerebral haemorrhage (ICH) model mice received slow-release buprenorphine (1 mg/kg), administered subcutaneously. A burr hole was then made 2.2 mm to the right of bregma using a 0.9-mm surgical drill bit (Fine Science Tools, USA) attached to a Dremel (Robert Bosch Tool Co., USA). A 2 μl glass Hamilton syringe, with a 25-G blunt needle (Harvard Apparatus, USA) containing collagenase (from *Clostridium histolyticum*, Sigma, USA) and diluted in normal saline, was mounted in a computer-controlled autoinjector apparatus and advanced at a rate of 1 mm/min through the burr hole to a depth of 1.6 mm, targeting the S1FL region of the cortex. The needle was allowed to dwell in the brain for 1 min to allow the tissue to relax along the needle path, and then a total of 0.4 μl of the diluted collagenase (0.075 units) was deposited into the brain at a rate of 0.2 μl/min. Following the deposition of the collagenase, the needle was allowed to dwell for 10 min to allow time for the collagenase to disperse from the injection location without exiting the brain via the needle tract, and then the needle was retracted at a rate of 1 mm/min. After procedures, marcaine (2 mg/kg, administered at 1.67 mg/ml in saline) was applied to the open incision site prior to suturing. ICH animals were maintained for 1 h to 1 day post-ICH.

Animals were allowed to recover in single-animal cages. At predetermined post-stroke time points (or at predetermined ages matching post-stroke subjects for controls), animals were euthanized by heavily anaesthetizing with 5% isoflurane followed by decapitation and immediately freezing the heads in liquid nitrogen to preserve the *in situ* state of the brain.

All animal work was conducted with approval from the University of Saskatchewan's Animal Research Ethics Board and carried out in accordance with the Canadian Council on Animal Care guidelines for humane animal use.

### Brain homogenization and synaptosomal isolation

Immediately prior to synaptosomal preparations, the 4°C Percoll stock solution was filtered through a Millipore AP15 filter to remove aggregated Percoll particles and then gradient solutions containing 3%, 10%, 15%, and 23% Percoll were prepared following published methods [28].

Seventeen-week-old male Balb/C mice (Charles River) were heavily anaesthetized with 5% isoflurane and then euthanized by cervical dislocation. The brain was removed expeditiously and placed in a 4°C isotonic buffer solution. The brain was then cut into smaller fragments and homogenized in a minimal volume of cold isotonic buffer solution. The homogenate was then centrifuged at 3600 rpm for 5 min to separate cellular debris. The supernatant was diluted in 5-ml homogenizing buffer and applied to the top of the Percoll gradient tubes. The tubes were centrifuged at 20 000 rpm (∼31 000 *g*) at 4°C in a fixed angle JA-25.50 rotor for 5 min to separate the components of the supernatant along the Percoll gradient. Of the five fractions generated, fraction 4 contained a mixture of synaptosomes and mitochondria.

A portion of whole brain tissue, the pellet, and ∼300 ml of the supernatant from the homogenate as well as a portion of fraction 4 from the Percoll gradient tube were applied to metal-free Nunc Thermanox plastic coverslips (Thermo Fisher Scientific Inc., USA) and allowed to air-dry. Small volumes of fractions 3, 4, and 5 were applied to separate Ni-grids and allowed to air-dry. Samples on Ni-grids were coated with phosphotungstate to increase X-ray contrast prior to transmission electron microscopy (TEM) data collection.

### Transmission electron microscopy and energy-dispersive X-ray spectroscopy

Imaging experiments were performed on a Hitachi HT7700 transmission electron microscope (TEM; Hitachi Ltd, Japan). Synaptosomes were identified visually and selected for energy-dispersive X-ray (EDX) spectroscopy. EDX was performed using a 100-keV excitation source and a 500-nm spot size focused on the centre of individual synaptosomes. EDX data were collected with a Bruker Nano silicon drift detector over an energy range of 100–80 000 eV.

### Synchrotron-based bulk X-ray fluorescence spectroscopy

Thermanox plastic coverslips containing whole brain, the pellet or supernatant from the brain homogenate preparation, or a volume from fraction 4 of the Percoll gradient were mounted in the beam at the IDEAS beamline at the Canadian Light Source synchrotron. Data were collected with the storage ring operating at 2.9 GeV and between 190- and 240-mA ring current. The beamline was equipped with a Ge(220) double crystal monochromator, which produced a vertically collimated beam. The incident energy was tuned to 13 450 eV and a beam spot of 2 mm × 6 mm was selected using vertical and horizontal slits. X-ray fluorescence data were collected in air over an energy range of ∼2500–20 000 eV using a Ketek Analytical X-Ray Acquisition System—Modular silicon drift detector (Ketek Gmbh, Germany) with an XIA DXP pulse processor (XIA LLC, USA). The excitation beam spot was ∼1 μm, as shown in Supplementary Fig. S1.

### Preparation of thin tissue sections for imaging experiments

Frozen brains were mounted in the cryostat at −18°C for cryosectioning. Fresh low-profile polytetrafluoroethylene-coated microtome blades were employed for sectioning each new brain to avoid trace metal contamination (Leica 819 blades: Leica Biosystems Inc., USA). Adjacent tissue sections were acquired for histology, XFI, and FTIR imaging. XFI sections were prepared at 30 μm thickness and mounted on Nunc Thermanox coverslips (Thermo Fisher Scientific Inc.) and allowed to dry before analysis. FTIR imaging samples were prepared at 10 μm thickness and mounted on CaF_2_ discs and stored in a −80°C freezer until data collection. No buffers, washes, or fixatives were employed in the preparation of brain sections in order to preserve the *in situ* state of the tissue.

### Preparation of whole body sections for imaging experiments

To produce a whole body section (shown in Fig. 1C), an 11-week-old female wild-type C57Bl/6 mouse (Jackson labs) was euthanized using 5% isoflurane, the whole carcass was washed gently, and embedded in 2.5% carboxymethylcellulose (Sigma, USA) using dry ice and 2-methylbutane following established methods [29]. A Leica CM3600 XP Cryo-macrotome (Leica Microsystems, Wetzlar, Germany) set at −20°C was used to prepare whole body sections (see Supplementary Fig. S2), which were cryosectioned at 30 μm thickness and mounted onto Kapton tape (Uline, Canada). Sections were desiccated at −20°C for 72 h, and then stored at room temperature until XFI imaging.

### Histology and optical microscopy

Tissues for histological staining were prepared at 14 μm thickness and mounted on Superfrost Plus microscope slides (Fisher Scientific, USA) and stored at −80°C. In some instances, the thicker tissue sections from XFI experiments were stained after XFI data collection. Sections (mounted on Thermanox plastic) were fixed with 4% buffered paraformaldehyde, rinsed with 1× phosphate-buffered saline, and then stained with hematoxylin and eosin (H&E). The stained tissues were imaged using the Aperio Virtual Microscopy system (Leica Microsystems GmbH, Germany) with a 20 × objective lens. Scanned data were extracted using the Aperio ImageScope software (Leica Microsystems GmbH, Germany).

### Fourier transform infrared spectroscopic imaging

Data were collected at the Mid-IR beamline at the Canadian Light Source synchrotron, using an Agilent Cary 760 spectrometer and Cary 620 Microscope, setup to operate in ‘off-line’ mode (non-synchrotron-based source), with a global serving as a broad-spectrum thermal source of infrared photons. The FTIR microscope is fitted with a 15× objective (0.62 numerical aperture) and data were collected from 900–3900 cm^−1^ in transmission mode. The detector is a liquid N_2_-cooled focal plane array that provides an effective field of view of 700 μm × 700 μm over an area of 128 ×* *128 pixels—with a pixel resolution of ∼5.5 μm. With 2 × 2 pixel binning, this provides a 64 × 64 pixel image for each snapshot of the sample. Mosaic maps of the sample are acquired tile-by-tile, acquiring 16 scans per tile (64 scans are averaged for the off-sample background from the CaF_2_ substrate). The imaging protocol employed provides an effective pixel size of ∼11 μm. Prior to each scan a tiled mosaic optical image of the sample was acquired, which aids subsequent image registration and fiducial landmarking. The Thermanox plastic used for mounting XFI sections is not compatible with FTIR imaging, similarly, the IR transparent CaF_2_ discs are not compatible with XFI; therefore, book-matched adjacent sections are collected where possible for comparison between techniques.

### Synchrotron-based X-ray fluorescence imaging data collection

Data were acquired from beamlines 7-2 and 14-3 at the Stanford Synchrotron Radiation Lightsource (SSRL), with the Stanford Positron Electron Asymmetric Ring (SPEAR3) operating at 3 GeV and a ring current of 450–500 mA, maintained by running in top-up mode. Beamline 7-2 is equipped with a 20-pole 2-T wiggler, using a liquid N_2_-cooled Si(111) sagittal focusing double-crystal monochromator (operating in the ϕ = 0° orientation) and an Rh-coated vertically focusing flat bent mirror. A microfocused beam was achieved using an aperture downstream of the I_0_ ion chamber, followed by a microfocusing polycapillary to achieve a spot size at the sample of ∼30 μm × 30 μm, with the sample mounted at 45° to the incident beam. Data from 7-2 were collected with a 4-element silicon drift fluorescence detector, using 200-ms dwell time per point, a 30 μm step size, and an incident energy of 13 450 eV. Acquired data at each pixel were normalized to the intensity of the upstream I_0_ ion chamber containing N_2(g)_. Prior to each XFI scan on beamline 7-2, a high-resolution optical image of the sample was acquired to aid image registration. Beamline 14-3 is a bending magnet side station, with a Ni-coated flat bent vertically collimating mirror, a Ni-coated bent toroidal mirror, and a water-cooled Si(111) double-crystal monochromator (ϕ = 0°). The beam was focused to ∼5 μm with a Kirkpatrick–Baez mirror pair with the sample mounted at 45° within an enclosed He_(g)_-filled chamber. Data from 14-3 were collected on a 7-element silicon drift fluorescence detector, using 100 ms dwell time per point, a 30 μm step size, and an incident energy of 2240 eV. Acquired data at each pixel were normalized to the intensity of the upstream I_0_ ion chamber containing He_(g)_.

Additional synchrotron-based XFI data were collected on the BioXAS-Imaging beamline at the canadian light source (CLS). During data collection the CLS storage ring operated at 2.9 GeV, with a ring current of 220 mA maintained in top-up mode. The BioXAS-Imaging beamline is equipped with an in-vacuum 1 T undulator, an Rh-coated collimating harmonic rejection mirror upstream of the Si(111) double-crystal monochromator, followed by a second Rh-coated focusing mirror. The beamline was operated in ‘macro’ mode with a slit assembly used to deliver a nominal beam spot size of 30 μm × 30 μm. Samples were mounted at 45° to the incident beam with a 4-element silicon drift fluorescence detector positioned at 90° to the beam. Brain maps were collected using a 100 ms dwell time per point and a 30 μm step size, while whole body sections were collected using a 60 ms dwell time with an 80 μm step size and a 0.5 mm Al filter upstream to attenuate the incident beam. Data were acquired with an incident energy of 13 450 eV and data at each pixel were normalized to the intensity of an upstream ion chamber containing N_2(g)_.

### AttoMap 310 X-ray fluorescence microscopy

XFI data were also acquired with an AttoMap™ X-ray Fluorescence Microscope, model 310 (Sigray, USA). A SiC target was used as the source of incident X-rays, which were directed onto the sample using a Sigray mirror lens focusing optic to generate a pencil beam 20 μm in size. Data were acquired under vacuum to improve detection of low-energy X-ray fluorescence signals with a silicon drift detector. Data were acquired with a 30 μm pixel size and a 2 s dwell time per pixel, while the remaining data were collected at a pixel resolution of 22.5 μm at a dwell time of 0.5 s. Prior to data collection, a tiled mosaic optical image of the sample was acquired for image registration and fiducial landmarking. Scan setup used the AttoMapXRF Micro X-ray Fluorescence software v0.0.39.2 (Sigray, USA).

### Data reduction and processing

XFI data from SSRL beamline 7-2 and 14-3, as well as CLS BioXAS-Imaging and AttoMap data, were processed using Sam's Microprobe Analysis Toolkit (SMAK), v3.0.9 [30]. Peak-fitting was performed for each X-ray fluorescence spectrum acquired in order to obtain more accurate data for low-intensity signals and to aid deconvolution of spectral regions with overlapping emission peaks (i.e. the Kβ and Ca Kα) [4]. Additional peak-fitting for bulk X-ray absorption spectroscopy (XAS) data obtained from TEM specimens was performed with the software application Peakaboo v5.5 [31].

Supplemental FTIR imaging data are processed with Quasar v1.9.2 [32] following established methods [33]. Biomarker and biomolecule maps are generated by integrating the area under the curve of the FTIR spectrum—with 1610–1690 cm^−1^ corresponding to the amide I region (protein), 1715–1755 cm^−1^ corresponding to the ν(C=O) band of lipid esters, and 1416–1475 cm^−1^ corresponding to the δ(CH_2_) mode arising predominantly from lipid tails.

Raw data were extracted from XFI and FTIR imaging data files using the in-house program datpush (v3.0) and visualizations were rendered in Fiji [34], employing the physics.lut lookup table for XFI data (available in Fiji), and the bbgy.lut lookup colour table for FTIR data (contained in reference 33).

## Results and discussion

### Overview of elemental composition of brain tissue

Figure 1A depicts isolation of brain tissue for bulk X-ray fluorescence characterization. Analysis of homogenate obviously abrogates the potential to appreciate the natural distribution of elements in the brain, but presents an opportunity to obtain an at-a-glance survey of a broad range of elements. Figure 1B shows the X-ray fluorescence spectrum collected from a portion of intact grey matter (whole brain) along with the pellet and supernatant obtained after centrifugation of the brain homogenate. Figure 1B demonstrates that each of these preparations contains a similar complement of elements. Homogenate is not only amenable to a broad range of experimental characterization techniques beyond X-ray fluorescence spectroscopy, but it can be subjected to further purification protocols to isolate specific components. For example, the isolation of a range of components from the homogenate is depicted in Fig. 1A, where a Percoll gradient is used to separate the mixture of suspended membrane components and subcellular structures (including synaptosomes and mitochondria) from the supernatant. A representative TEM image of an isolated synaptosome is shown in the inset image in Fig. 1A, mounted on a Ni TEM grid. The synaptosome labelled with ‘EDX target’ in Fig. 1A was selected for further X-ray excitation, and its emission spectrum is shown in the second panel in Fig. 1B. The isolated synaptosomes all demonstrate elevated Cu Kα and Kβ fluorescence above the background (acquired from a region only containing Percoll beads), and the Cu signal is also discernable above the relatively high Ni background fluorescence arising from the Ni grid used to mount the specimen (see Supplementary Fig. S1). Along with Cu-containing metalloproteins and Cu within mitochondria [35], this synaptosomal fraction of Cu contributes to a portion of the background Cu fluorescence within brain tissue.

Moving beyond the bulk tissue analysis, elemental mapping affords the ability to visualize the distribution of elements at their normal biological levels *in situ*. The selected element maps in Fig. 1C demonstrate the high background fluorescence signal arising from bone, which is significantly more dense and contains greatly elevated Ca content compared with soft tissue. The presence of bone can readily saturate EDX fluorescence detectors, deteriorating count rates and generating aberrant artefacts in the spectrum [4]; however, attenuation of the incident beam can aid in the reduction in overall count rates from such regions of the sample, although this also reduces the signal-to-noise in soft tissue regions and may make detection of ultra-low trace elements more challenging. In each of the elemental maps in Fig. 1C, the anatomic distribution and variation in concentration are apparent. The brain is high in K throughout, as K^+^ is essential for neurons to maintain their membrane potential. Compared with hardened bone, most tissues are relatively low in Ca. Likewise, Cu is also maintained at low levels in most tissues, although elevated Cu is discernable in periventricular regions of the brain. Zn is present at low levels throughout the brain and displays variation in concentration with anatomic location and with cell type, making it more useful than other elements for identifying brain structures through the elemental maps alone.

### Key elements of the neurometallome

**Figure 2.**
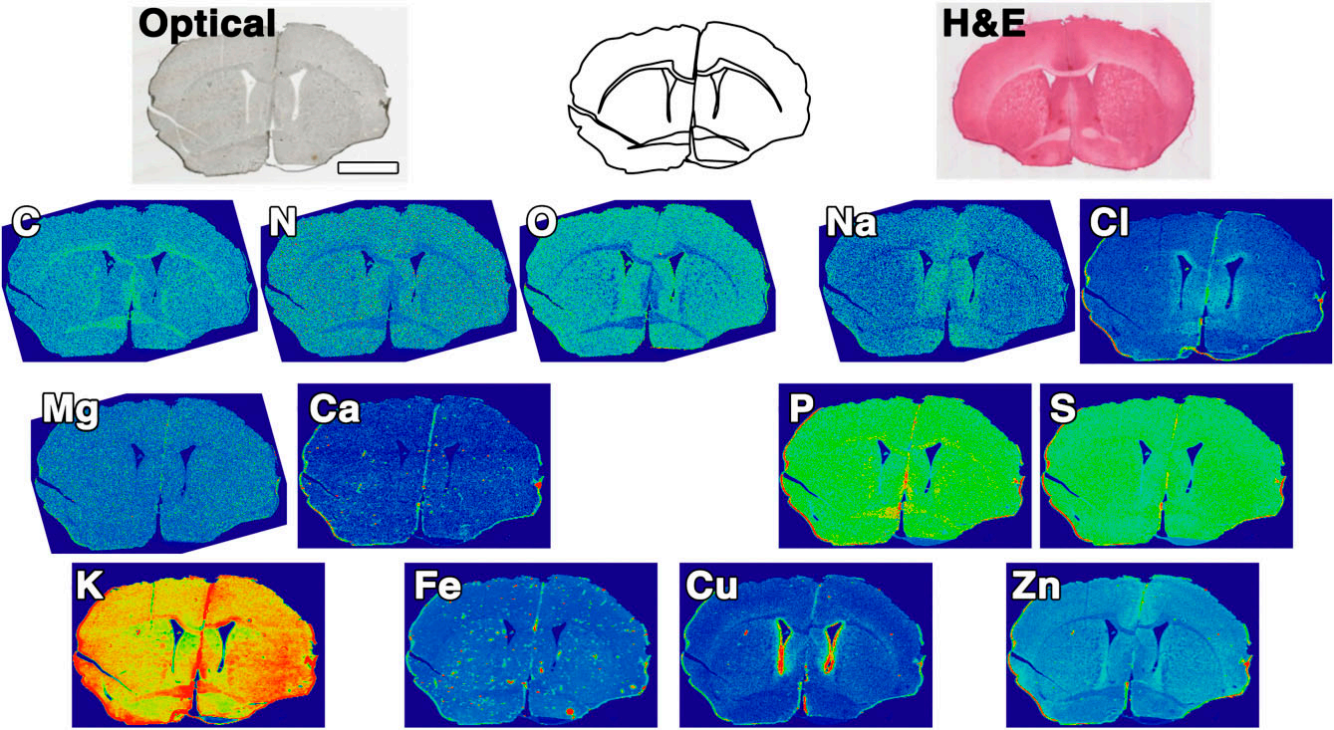
Coronal brain sections for a representative healthy control mouse brain (11-week-old female). The optical microscopy image is shown for reference along with the schematic diagram of the tissue. The H&E staining was performed after XFI data collection. Scale bar = 2 mm.

Elemental mapping is useful for identifying specialized regions of the brain and particular cell-type populations—such as the Zn-rich dentate gyrus [36] (which can be appreciated in the sagittal view in Fig. 1C), the high Cu content of the periventricular regions of the ventricles (Figs 1C and 2), or high Fe signals in blood vessels and inflammatory cells [37] (Figs 1C and 2). Figure 2 presents a summary of the most abundant elements of the neurometallome, as well as additional elements that are particularly elevated in the tissue.

The light non-metal elements C, N, and O are ubiquitous throughout the brain, with the only significant intensity changes emphasizing differences in tissue density, such as between grey and white matter regions (see Fig. 2). It is worth noting that the tissue is effectively dehydrated at the time of imaging and therefore the O Kα fluorescence in Fig. 2 predominantly arises from biomolecules and low-molecular-weight metabolites in the tissue.

Both Na and Cl (Fig. 2) are commonly encountered ions in biological tissues and are expected to be particularly elevated in the lumen of blood vessels (see concentrations in Table 1) as well as in periventricular regions of tissue. Blood vessel identification in the Na or Cl maps is challenging, and more readily done in Fe maps. The concentrations of Na and Cl are controlled by a combination of passive and active transport as well as paracellular diffusion at the capillary level in the brain.

Elsewhere in the body Ca^2+^ in the blood is in slow equilibrium with bone, but in the brain the barrier systems actively limit the brain to lower Ca^2+^ levels overall, as highlighted in the Ca levels in Table 1. Compared with blood, Mg^2+^ is maintained at a higher concentration in the brain (fluid compartments and the brain parenchyma), whereas the brain parenchyma contains significantly lower levels of Ca^2+^ compared to blood. Because Ca^2+^ plays a central role in cellular signalling events in the brain, the level of labile Ca^2+^ (sometimes referred to as ‘free’ Ca) in neurons has been reported to be ∼6 × 10^−8^ M [12]. The distribution of Ca^2+^ (atomic number, *Z*, = 20) is relatively uniform throughout the brain (Fig. 2) and is at a significantly lower concentration compared to K^+^ (*Z *= 19), for example. X-ray fluorescence emission can potentially lead to misleadingly high apparent Ca Kα fluorescence because the K Kβ emission line significantly overlaps. In brain tissue, where the concentration of K^+^ is two orders of magnitude higher than Ca^2+^, the relatively high K Kβ peak (despite being only ∼10% the amplitude of the K Kα peak) can be mistakenly interpreted as high Ca signal when binning photon counts. The best method for data processing and reduction of XFI data where peak overlap occurs is to deconvolute such overlapping contributions by performing peak-fitting, as previously described [4].

While not metals, the abundance of both P and S in brain tissue shown in Fig. 2 merits comment. Both elements play specialized structural, chemical, and signalling roles in biomolecules and bioinorganic complexes in biological systems. Moreover, P and S are useful elements at the organ level to identify tissue regions and signs of metabolic dysfunction or injury [38]. Both elements are ubiquitous and, like C, N, and O, are incorporated into many biomolecules. P is found in ATP and related metabolites, in the phosphodiester backbone of DNA, as free phosphate as well as in kinase-tagged proteins, and in the headgroups of phospholipids, as examples. Similarly for S, this is a critical element in cystine and methionine and is therefore expected anywhere protein levels or the free amino acids are abundant, in sulpholipids, is incorporated into 3′-phosphoadenosine-5′-phosphosulphate, and other low-molecular-weight sulphur species. Importantly, subtle differences in the relative intensities for P and S can aid differentiation of tissue types (i.e. those in Fig. 2) as well as help aid identification of regions of tissue damage or changes in metabolism. The broad ubiquity of P and S, however, also hinders more refined interpretation of XFI data, as it can be challenging to pin down the underlying biochemical changes in the tissue that are driving alterations in total P or S concentrations.

The brain is a privileged organ in regard to the tight regulation of elements, particularly potassium. High concentrations are necessary to maintain the electrochemical potential across the neuronal membrane, with relatively high concentrations also found in supporting astrocytes (Table 1), which play a role in helping to clear extracellular K^+^ after depolarization. The distribution and concentration of K^+^ in tissue are also tied to the transport of other ions through coupled transport (Fig. 2), such as via the Na–K–2Cl cotransporter (KNCC1), for example [39].

The Fe distribution map in Fig. 2 demonstrates the typical heterogeneous distribution of this element in the brain. A low-level background of Fe is present throughout all tissues, while regions of high Fe (orders of magnitude more highly concentrated) correspond to blood vessels and to surveilling microglial cells in the parenchyma or macrophages from peripheral blood. Fe is also a common contaminant that may arise from environmental sources (i.e. dust) as well as dispersed frozen blood fragments that get dislodged from the frozen sample and re-deposited on the tissue during cryosectioning. Fe in contaminating dirt and dust on samples can often be differentiated from biological Fe due to the exceptionally high concentration of Fe and elevated concentration of other elements, such as Ti, Ni, or Cr, that may be co-localized with the Fe signal. The concentration and size differences in Fe distributions can be exploited in automated segmentation routines to identify blood vessels or ferritin-containing cells, for example. Blood vessels can be challenging to identify by eye in the busy Fe maps, but tend to be on the order of many tens of microns in size and have an intermediate Fe level that is between the Fe background and the Fe level in microglial cells. In coronal sections some blood vessels may be visible in the cross section in the midbrain region along with occasionally visible penetrating arteries that traverse the cortex, which are seen in the longitudinal cross section. Microglial cells tend to contain remarkably high levels of biological Fe, which aids their identification against the low Fe background in the brain and can even be differentiated in regions of haemorrhage [40]. The ferritin can contain up to 4500 atoms of Fe and the protein shell is 12 nm in diameter (measured from the deposited heavy-chain ferritin structure 8DNP) [41] and typically occupies a single pixel in image maps at the micron pixel scale [40, 42]. XAS of these small, high-intensity, Fe signals can confirm the cellular identity of these pixels as the element will be in the ferrihydrite form [40]. The high concentration of Fe in these cells is one reason Fe is so commonly correlated with inflammatory conditions and disease states [43–46]. Much work remains in this area to differentiate whether Fe plays a direct causative role in onset of many diseases and progression of disease, or whether its presence is merely consequential to the presence of macrophages. Importantly, in this regard however, apoptosis of ferritin-containing cells leads to ferritin release and loss of the ferritin protein coat, but does not disrupt the ferrihydrite core, which results in deposition of the core as hemosiderin, which can be retained for prolonged periods [47, 48].

Cu is cited as one of the most tightly regulated elements, with the published estimate of one free (labile) atom of Cu per cell [49]. The term ‘free’ in the context of metal ions is not chemically specific, but meant to connote a labile state that allows the metal to be taken up by other biomolecules or partake in side reactions. Trace elements like Cu, Fe, and Zn are common essential elements for the growth and reproduction of microbes, which is the reason the peripheral immune system sequesters metals as part of its immune response, aside from inflammation [50, 51]. Additionally, the impetus for biological systems to maintain tight control of metal levels, Cu in particular, is the proclivity of metals to partake in deleterious Fenton chemistry [52], where labile metals can readily catalyse the production of free radicals—with Cu being notably efficient in this regard [53]. Cu is found at trace levels throughout the brain, although unlike the synaptosomal fraction in Fig. 1, the highest levels are found in periventricular regions (Fig. 2) where it is stored in glial cells within the subependymal zone [54–56]. This subependymal Cu predominantly exists as a combination of Cu^2+^, in an environment similar to superoxide dismutase, and Cu^+^, possibly bound to metallothionein [54]. Loss of essential Cu in oligodendrocytes has been implicated as a causative step in the demyelination observed in mouse models following treatment with cuprizone [57, 58].

Zn is ubiquitous in the brain, with sufficient variability in concentration depending on neuroanatomic location and cell type that it is possible to use Zn maps to aid image segmentation and anatomic landmarking (Figs 1C and 2).

### Stroke leads to profound dysregulation of the neurometallome

**Figure 3.**
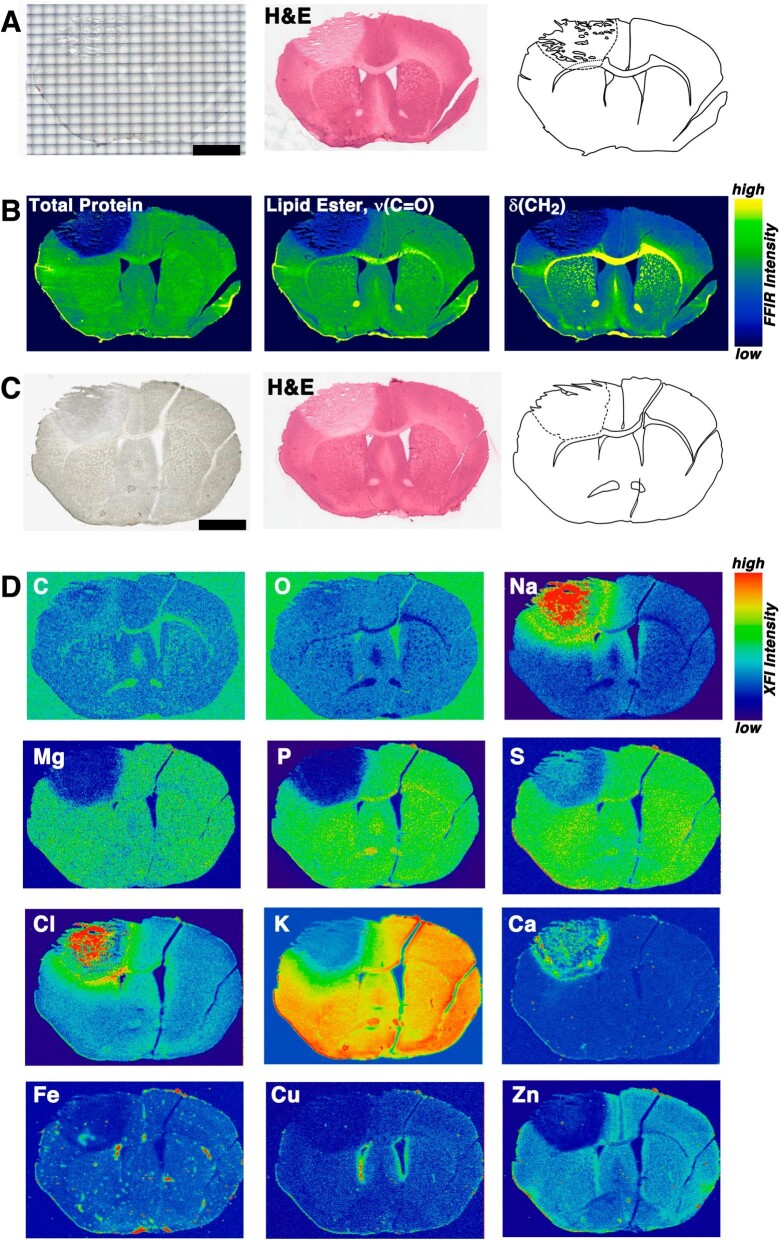
Representative example of ischaemic stroke in a 72-h post-photothrombotic mouse model. (A) Tissue section used for FTIR imaging, showing the optical image, H&E staining, and schematic diagram. (B) FTIR imaging of key biomarkers. (C) Tissue section used for XFI. (D) XFI data for the key elements in brain tissue. XFI data were collected with a combination of synchrotron radiation for heavier elements, while lighter elements (*Z *< 16) were acquired with an AttoMap 310 (Sigray, USA). Scale bar = 2 mm.

Elemental mapping of post-stroke brain tissue exquisitely demonstrates dysregulation of the key elements in the pathophysiology of stroke. We have recently demonstrated that at very early post-stroke time points (5 min post-reperfusion after a 30-min MCAO) that K^+^ is already noticeably dysregulated, leading to reduction in total K in the affected tissue [59]. Over the course of the next 60+ min, the total K^+^ continues to decline [59], which is only possible if it is diffusing out of the tissue, as there is no detectable elevation in K content in peripheral tissue [59, 60]. In this same duration the lesion continues to expand and the dysregulation of additional elements becomes evident [59]. We have observed that by 72 h post-stroke the lesion has essentially stopped expanding [60]. In the 72-h ischaemic stroke example in Fig. 3, the stroke lesion is readily discernable in all panels. Figure 3A shows the tissue used for FTIR imaging, with the optical microscopy image of the coronal section (collected through the centre of the photothrombotic stroke lesion) as well as the H&E-stained tissue and the schematic outline of the tissue. Figure 3B shows some relevant biomarker maps from FTIR imaging, acquired prior to H&E staining, and demonstrates the total protein content and two different lipid absorption bands, which show a clearly demarcated border for the ischaemic lesion (for further detail on FTIR imaging of brain tissue and stroke, see reference 33). Figure 3C shows the tissue section used for XFI, collected adjacent to the section in panel A, along with the H&E and schematic diagrams of the section. Figure 3D shows all of the most significantly affected elements due to stroke in the specimen, excluding those elements that are at or below the detection limits of the scan parameters (most notably Mn and Se are not shown). Both C and O maps show subtle differences in intensity in the lesion, which is likely due to underlying differences in density compared to bulk tissue. Both Na and Cl are elevated in the lesion and display similar distribution patterns in the bordering peri-infarct zone. The elevated Na and Cl, along with K, are notable in that they represent the largest areas of metabolic dysregulation of all elements. In contrast, the other elements have much better-defined borders that demarcate the lesion core from intact tissue. It should be noted that although the elevated distribution of Na and Cl appears to extend through the medial region between the ventricles and into the adjacent hemisphere beyond the contralateral ventricle, these are regions of normal elevation for these elements and represent regions of Na and Cl trafficking. The transition metals, Fe, Cu, and Zn, are also reduced in the lesion core, most likely due to the reduced pH and necrotic changes [61–63]. Transition metals are particularly sensitive to reduced pH conditions, which tend to destabilize all but the most tightly bound metal ions, generating labile and readily diffusible low-molecular-weight metal ions. The elevated Ca in the lesion is remarkable in that, like Na and Cl, it represents a significant excess beyond what can be accounted for from brain tissue. We hypothesize that the breakdown of the blood–brain barrier and diffusion of high concentrations of these ions from blood are the underlying cause and source of the vastly elevated levels of these elements in particular. The distribution of Ca, however, displays a more varied and patchy distribution in the lesion and is due to a drying artefact, where this ion tends to aggregate and form microcrystals as the tissue dries after sectioning [60]. Some regions of the ischaemic infarct may show elevated Fe, such as the lateral inferior border of the Fe map in Fig. 3D. This elevated Fe content is the result of haemorrhagic transformation of the ischaemic lesion, which occurs in ∼15% of all ischaemic stroke cases [64]. We note approximately the same incidence rate of haemorrhagic transformation in the photothrombotic and MCAO models in our mouse models [40, 60]. The presence of haemorrhagic transformation of ischaemic stroke is a potential confounding variable for animal models in the stroke literature—particularly for early post-haemorrhage time points or minimal brain bleeds, which may not present visual clues during tissue sectioning as may appear unremarkable from an ischaemic lesion with conventional stains (i.e. H&E stain in Fig. 3).

**Figure 4.**
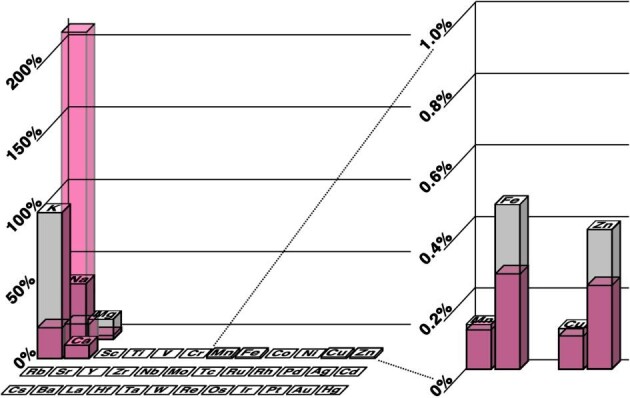
Concentration of elements relative to [K] in cortical grey matter. Typical metal levels are shown as grey columns with their element symbol, whereas 72-h post-ischaemic stroke levels are shown as a coloured overlay, highlighting the significant differences between each element. Summary of changes for each element, relative to its initial concentration: Na 558.9%, Mg 19.7%, K 23.5%, Ca 225.3%, Mn 93.1%, Fe 56.6%, Cu 83.0%, and Zn 60.6%. Regions of haemorrhage have a ∼1500% increase in Fe content due to Fe-rich erythrocyte intrusion into the brain parenchyma.

Figure 4 summarizes the relative changes in element levels in the brain, normalized to [K^+^], where solid columns indicate normal levels in grey matter and the shaded overlays indicate the altered concentrations at 72 h post-stroke in ischaemic stroke models.

**Figure 5.**
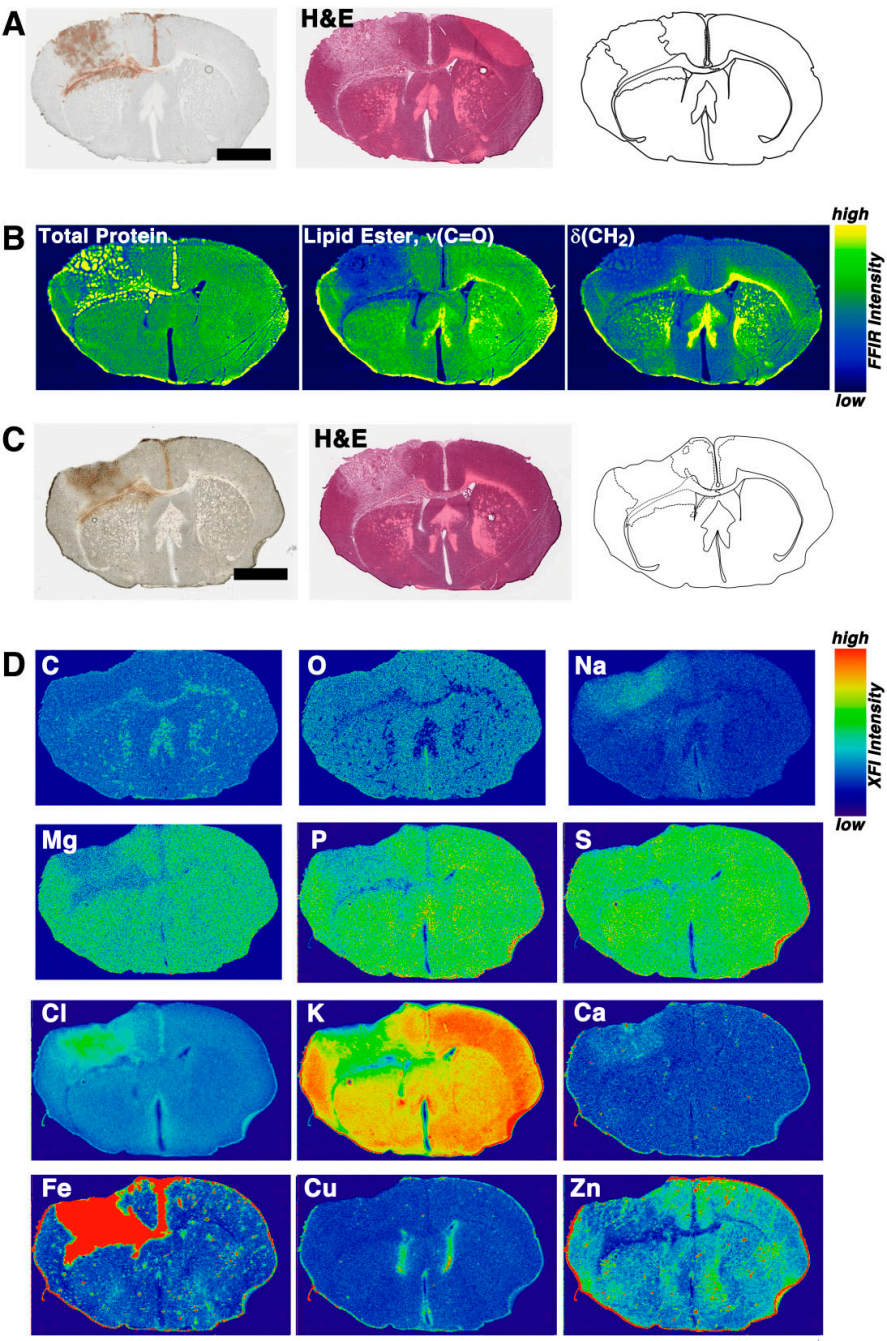
Representative example of ICH at 1 h post-injection of collagenase. (A) Tissue section used for FTIR imaging, showing the optical image, H&E staining, and schematic diagram. (B) FTIR imaging of key biomarkers. (C) Tissue section used for XFI. (D) XFI data for key elements in brain tissue, with bleeding evident in the cortex as well as along the midline sagittal sulcus and the border of the right lateral ventricle in the Fe map. XFI data were collected with a combination of synchrotron radiation for heavier elements, while lighter elements (*Z *< 16) were acquired with an AttoMap 310 (Sigray, USA). Scale bar = 2 mm.

For comparison with the typical ischaemic stroke example shown in Fig. 3, a model of induced ICH at 1 h post-haemorrhage onset is shown in Fig. 5. The haemorrhage is readily identifiable in the optical microscopy image in Fig. 5A, acquired before FTIR imaging. The H&E-stained tissue and the schematic outline are shown for reference. The soaking and rinsing steps required for the staining remove the majority of erythrocytes from the region of haemorrhage, making it appear similar to the ischaemic lesion in Fig 3A. The FTIR imaging (Fig. 5B) of this tissue shows significantly elevated protein content in the lesion, largely due to the protein-rich erythrocytes and albumin from blood [33]. The loss of lipid content is most identifiable in the lipid ester map, although the δ(CH_2_) map (lipid tails) is useful for identification of regions of white matter loss, such as the region of the corpus callosum along the inferior border of the lesion in Fig. 5B.

The adjacent tissue section for XFI (Fig. 5C) is imaged with optical microscopy and the H&E stained and schematic outline are shown for additional reference. The optical image again highlights the regions of tissue containing blood products. This ICH example in Fig. 5D shows more subtle differences in concentrations for most elements compared with Fig. 3D due to the significantly shorter post-onset duration in this example (1 h vs. 72 h post-stroke in Fig. 3). For example, the distributions of increased Na and Cl and reduced K closely mirror one another, with a relatively large border containing a gradient between the concentrations of the core and normal tissue (identical trends can be seen at 90 min post-reperfusion in the MCAO stroke model, Supplementary Fig. S3). Most other elements, however, demonstrate a smaller region of the affected tissue and less overall dysregulation at this time point. The most notable change relative to the ischaemic stroke example from Fig. 3 is the vastly elevated Fe signal throughout the affected tissue (Fe map in Fig. 5D). The Fe map is difficult to display due to the large dynamic range in Fe levels between the background and the haemorrhage (converting to a log-scale does not improve the rendering). The Fe map is also remarkable because the presence of elevated Fe is evident well beyond the haemorrhage borders that are apparent in the optical image in Fig. 5C, demonstrating the sensitivity of XFI for subtle, low-intensity, Fe diffusing into the peripheral tissue this early after haemorrhage onset.

The microglial cells responding to post-stroke inflammation bring with them their own complement of elements, including Fe in the form of ferritin [40] and astrocyte foot processes wall-off the core of the stroke lesion through formation of the glia limitans [65]. The walled-off ischaemic core represents irretrievably lost tissue without potential for functional recovery. The isolation of the core by the glia limitans serves as a barrier, in part, to protect surviving cells of the central nervous system outside this barrier, and to allow cells within the core to initiate restoration toward otherwise normal trace element levels in the tissue. The contribution of responding inflammatory cells and the dynamic changes in water and element (and metabolite) content during the acute and into the chronic post-stroke phase puts limits on the potential for functional recovery of surviving cells outside the lesion core.

## Conclusions

Our multimodal imaging results reveal the extent of ion dysregulation and altered metal content in injured brain regions after stroke onset. The prevailing dogma of stroke pathophysiology focuses on cell-level changes in energy, metabolism, and ion regulation. Indeed, these cellular-level events drive the underlying pathophysiological changes in stroke; however, the experiments summarized herein, and expanded upon elsewhere [40, 59, 60, 66, 67], highlight the significant organ-level changes that occur in the first days after stroke onset. The profound changes in the neurometallome that accompany stroke likely have significant consequences for the feasibility of many stroke treatments and the potential for meaningful functional recovery [68]. For example, the extreme reduction in total [K^+^], including in the tissue bordering the core of the lesion, likely has consequences for the efficiency of functional recovery in surviving neurons in this region. Even with prompt reperfusion and restoration of energy in the affected tissue, as modelled with a 30-min MCA occlusion, the surviving neurons therein may not be able to accumulate enough K^+^ to establish the requisite membrane potential for activity [69]. Related to this is the significant reduction in Mg^2+^ (Figs 3D and 5D). Biological activity of ATP relies on formation of an adduct with Mg^2+^, and it is this Mg–ATP complex that is most readily utilized by cells [70]. Therefore, notwithstanding the existing energy metabolism challenges for surviving neurons (such as the decoupling from supporting astrocytes [71]), the loss of Mg^2+^ and K^+^ presents practical challenges that must be overcome, beyond restoration of an energy supply. Additionally, there may be further limitations on the potential for functional recovery in the immediate post-stroke period. The propensity for the affected tissue to lose essential Cu and Zn may have deleterious functional consequences for synaptic transmission in vicinity of the lesion due to reduced synaptic Cu content [72, 73] and reduced Zn content for gluzinergic neurons [74, 75].

While regions of ischaemia are demonstrably low in Fe and Cu content, these regions of tissue are nevertheless highly susceptible to oxidative damage due to trace metal dysregulation [40]. The restoration of blood flow and O_2_ perfusion present well-known challenges to ischaemic tissue [76]. Even though the stroke lesion may contain reduced transition metals overall, only catalytic amounts of labile Fe or Cu are required to initiate ongoing deleterious Fenton chemistry and only a modicum of success has been found with metal chelators; however, post-treatment outcome did not improve vs. controls [77]. It remains unclear whether chelation may pose separate challenges for recovery of surviving cells already present in an environment with reduced essential metals. Interestingly, C57BL/6 mice appear to be exquisitely susceptible to demyelinating disease, which is hypothesized to arise through chelation of essential copper from supporting oligodendrocytes, and this may hinder repair and recovery of white matter in the early post-stroke phase [57]. Additionally, ischaemia damages mitochondria, which can also contribute to ongoing oxidative stress due to leaky electron transport chains [78], further complicating potential recovery.

The expanded view of the extent of dysfunction at the level of the neurometallome and the significant tissue changes in essential trace elements that are revealed with XFI demonstrate a more profound level of dysfunction than can be predicted from a cellular-level description of the physiological changes coinciding with ischaemic stroke alone. The neurometallome is uniquely challenged after stroke onset, and although the magnitude of these changes should give pause for consideration of the potential for functional recovery of cells in the vicinity of the dysregulation, these experiments also present an array of challenging targets for recovery of the neurometallome in future stroke treatments.

## Supplementary Material

mfae054_Supplemental_File

## Data Availability

The data underlying this article will be shared on reasonable request to the corresponding author.
