# Supplementary material for: X-ray fluorescence mapping of brain tissue reveals the profound extent of trace element dysregulation in stroke pathophysiology
Source: Metallomics. 2024 Nov 15;16(12):mfae054. doi: 10.1093/mtomcs/mfae054 (PMC11631071; doi:10.1093/mtomcs/mfae054)
Supplement: mfae054_Supplemental_File [file mfae054_supplemental_file.docx]

*For the Special Issue on Neurometallomics in Metallomics.*

SUPPORTING INFORMATION FOR

**X-ray Fluorescence Mapping of Brain Tissue Reveals the Profound Extent of Trace Element Dysregulation in Stroke Pathophysiology**

M. Jake Pushie,^a*^ Nicole J. Sylvain,^a^ Huishu Hou,^a^ Nicole Pendleton,^a^ Richard Wang,^b^

Liam Zimmermann,^b^ Maxwell Pally,^c^ Francisco S. Cayabyab,^a^ Lissa Peeling,^a^ Michael E Kelly^a*^

^a^ Department of Surgery, Division of Neurosurgery, College of Medicine, University of Saskatchewan, Saskatoon, SK, S7N 5E5 Canada

^b^ College of Medicine, University of Saskatchewan, Saskatoon, SK, S7N 5E5 Canada

^c^ College of Arts & Science, University of Saskatchewan, Saskatoon, SK, S7N 5E5 Canada

* Corresponding authors: jake.pushie@usask.ca; m.kelly@usask.ca

Department of Surgery

University of Saskatchewan

107 Wiggins Rd B419

Saskatoon, SK

S7N 0W8

CANADA

Ph: (306) 966-8641

Fax: (306) 966-8026


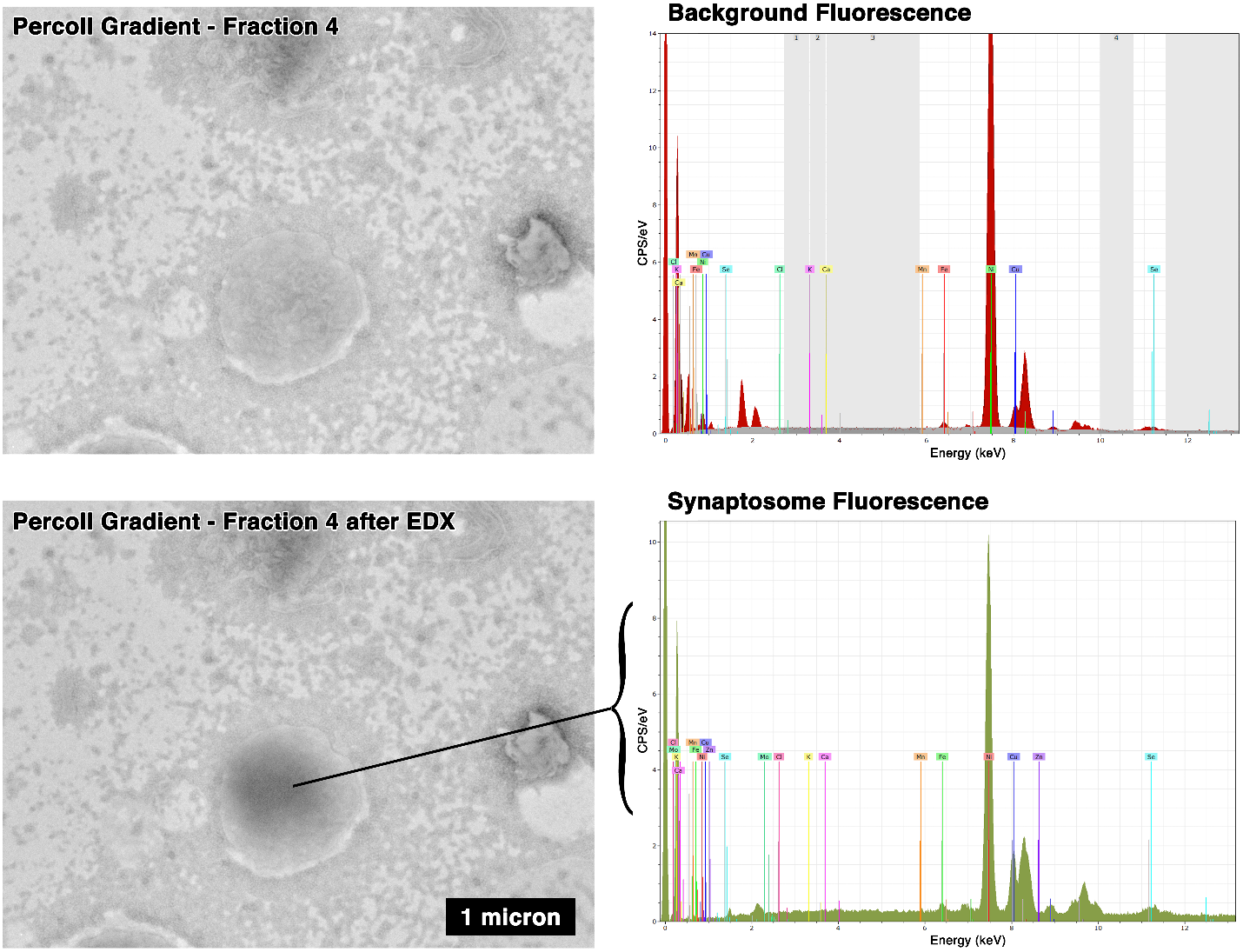


**Figure S1.** Transmission electron microscopy images of synaptosomal fraction after Percoll gradient centrifugation. Samples were mounted on a Ni-grid, which contributes to the X-ray fluorescence background (red spectrum, note low-level Cu Kα fluorescence). After excitation of a selected synaptosome the Cu Kα fluorescence is demonstrably higher than background (green spectrum). Photodamage of the interrogated spot is visible as a dark spot in the TEM image.


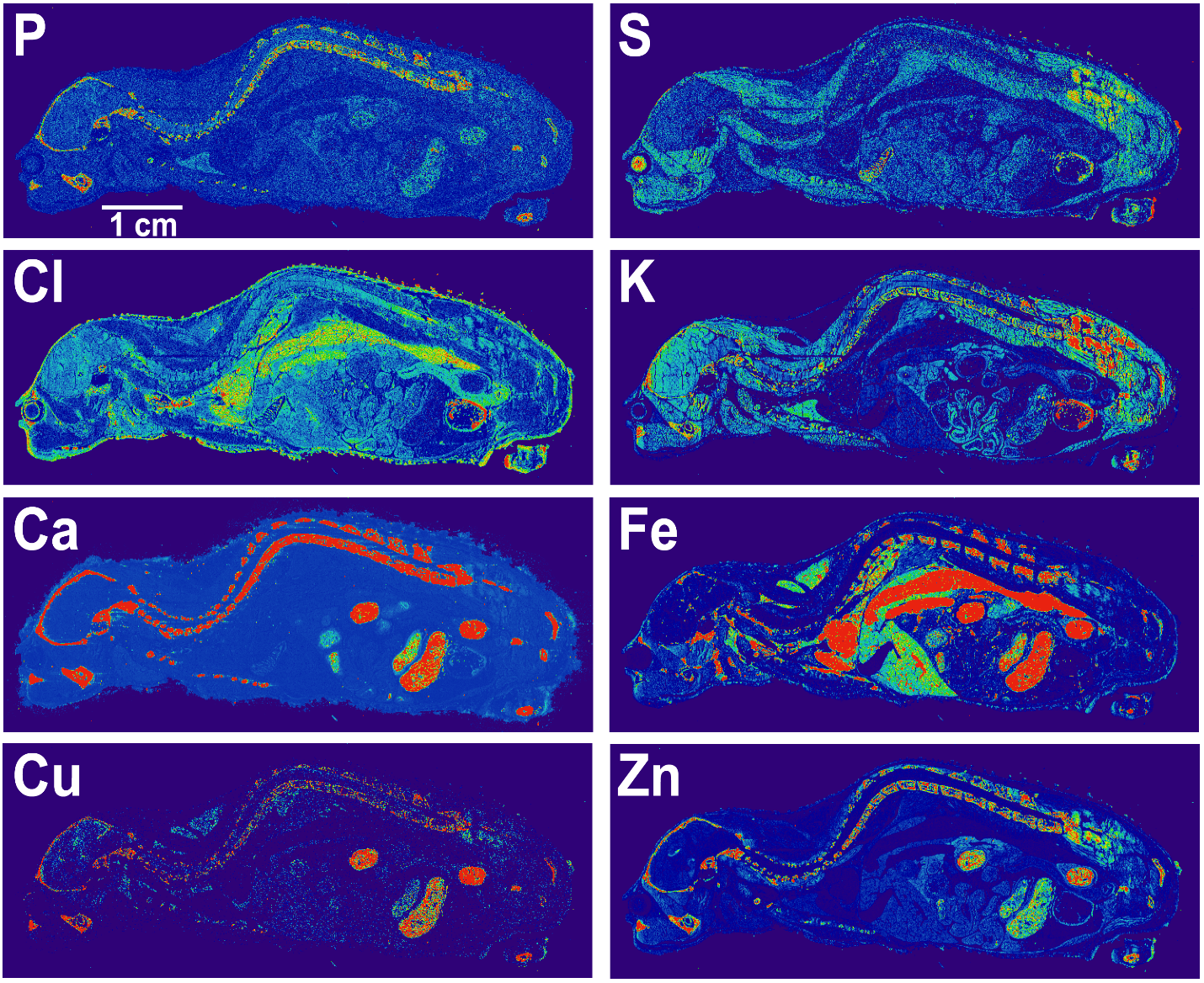


**Figure S2.** Sagittal view of a female C57/BL6 mouse. Tissue cryosectioned to 30 μm thickness. Data corresponds to lower pixel resolution survey scan of data shown in Figure 1C.


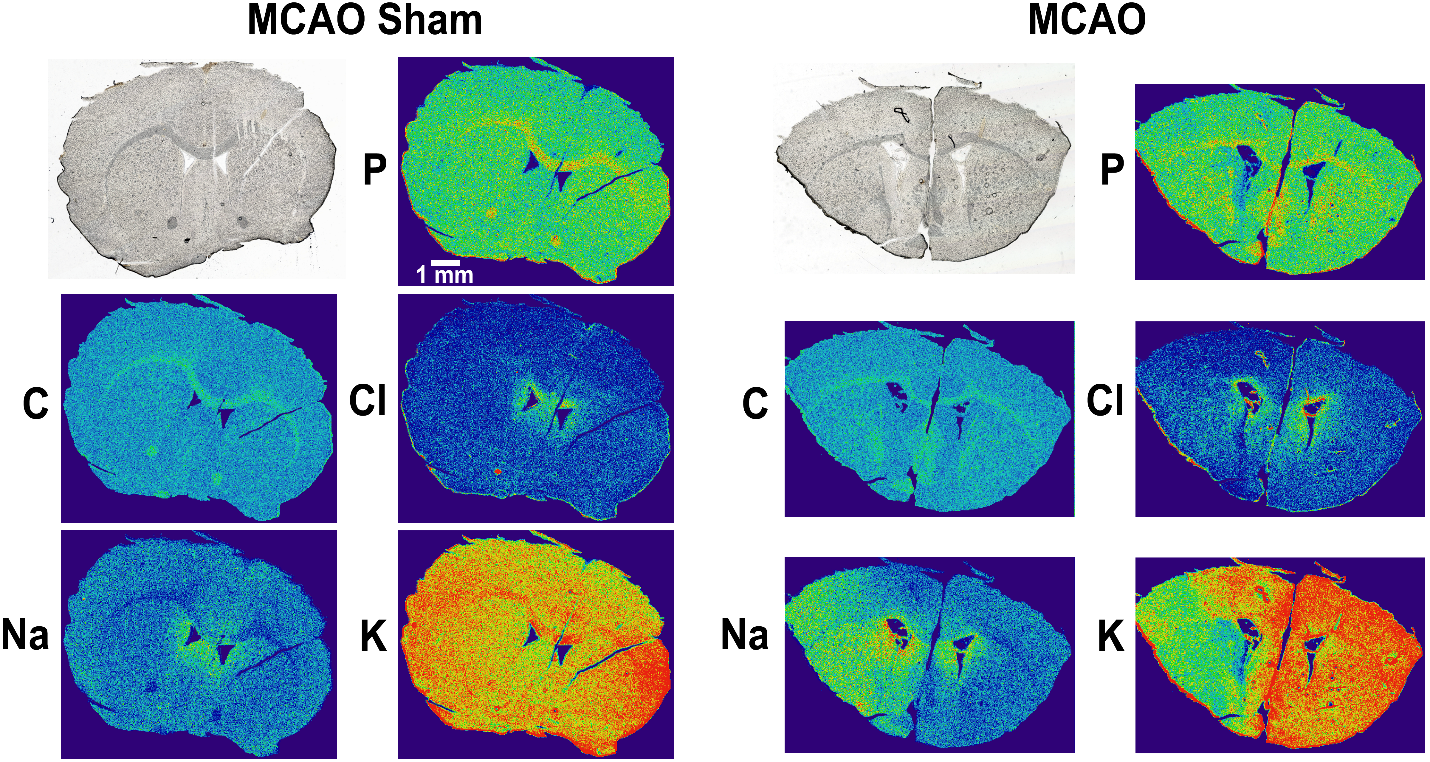


**Figure S3.** Representative element dysregulation from MCAO sham (control) and MCAO stroke model. MCAO model employed a 30-minute occlusion, tissue collected at 90-minutes post-reperfusion.
